# Whole blood viscosity is associated with extrahepatic metastases and survival in patients with hepatocellular carcinoma

**DOI:** 10.1371/journal.pone.0260311

**Published:** 2021-12-02

**Authors:** Ji Won Han, Pil Soo Sung, Jeong Won Jang, Jong Young Choi, Seung Kew Yoon

**Affiliations:** 1 The Catholic University Liver Research Center, College of Medicine, The Catholic University of Korea, Seoul, Republic of Korea; 2 Division of Gastroenterology and Hepatology, Department of Internal Medicine, College of Medicine, Seoul St. Mary’s Hospital, The Catholic University of Korea, Seoul, Republic of Korea; Nihon University School of Medicine, JAPAN

## Abstract

Whole blood viscosity (WBV) is increased in cancer patients and associated with the advanced stage with systemic metastases. However, relevance of WBV in hepatocellular carcinoma (HCC) remains unclear. This pilot study included a discovery cohort of 148 treatment-naïve HCC patients with preserved liver function, and a validation cohort of 33 treatment-experienced HCC patients with nivolumab. Systolic and diastolic WBV was measured using an automated scanning capillary tube viscometer at diagnosis or before the nivolumab treatment. Extrahepatic metastases were observed in 15 treatment-naïve patients (11.3%) at diagnosis. Portal vein tumor thrombosis (PVTT), tumor size, number of tumors, and systolic/diastolic WBV were factors associated with extrahepatic metastases. Systolic WBV and diastolic WBV were significantly increased in patients with metastases compared with patients without metastases. Multivariate logistic regression showed that high diastolic WBV > 16 cP was an independent factor associated with metastases. Notably, patients who developed extrahepatic metastases during the observation period among patients without metastases at diagnosis had higher diastolic WBV initially. Patients with high diastolic WBV had poor survival, and multivariate Cox regression analyses showed high diastolic WBV was an independent risk factor for poor survival with the Child-Pugh B7 and PVTT. High diastolic WBV also predicted poor survival in patients with low alpha-fetoprotein (AFP) and proteins induced by vitamin K antagonist-II (PIVKA-II) levels. In 33 nivolumab-treated patients, high diastolic WBV before the treatment was also tended to be associated with overall and progression-free survival. Our study is the first in which high WBV is associated with the distant metastases and survival in patients with HCC, but future prospective, large cohort studies are necessary to validate the results.

## Introduction

Hepatocellular carcinoma (HCC) is the sixth most common cancer worldwide and a representative cause of cancer-related death [1]. Innovative advances have been made in the treatment of HCCs, mainly due to the recent development of systemic agents besides sorafenib, such as lenvatinib, regorafenib, cabozantinib, and ramucirumab as well as combination regimens (e.g., atezolizumab plus bevacizumab) [2]. However, minimal development has been achieved in terms of HCC biomarkers although alpha-fetoprotein (AFP) and proteins induced by vitamin K antagonist-II (PIVKA-II) have been used for diagnosis and predicting clinical outcomes. Nevertheless, substantial number of HCC patients are negative for these tumor markers [3]. Thus, concomitant advances both in the treatment modality and development of biomarkers could help in establishing the proper strategy and possibly improve clinical outcomes of HCC.

Blood viscosity is defined as the stickiness of blood and determined based on the ratio of shear stress to shear rate. Because liver plays a central hemorheological role and synthesizes molecules associated with hemorheology [4, 5], the alteration of blood viscosity in human liver diseases has been studied, although the clinical relevance remain to be elucidated. In several studies, whole blood viscosity (WBV) was shown to be significantly decreased in patients with liver cirrhosis (LC) [4–7]. In contrast, patients with non-alcoholic fatty liver disease [8, 9] or chronic hepatitis B virus (HBV) infection [7] have higher WBV compared with controls. Notably, we previously showed that WBV measured using a new scanning capillary tube viscometer is inversely correlated with liver stiffness [10], indicating that WBV can be used as a biomarker in chronic liver diseases. Importantly, Child-Pugh score C and the history of hepatic encephalopathy were associated with significantly low blood viscosity levels [11].

Hemorheological alterations in malignancy have also been studied. In several studies, blood viscosity was increased in cancer patients compared with controls [12–14]. Blood viscosity positively correlated with the cancer stage [15] and was associated with metastases [12, 16], indicating blood viscosity might have a role as a biomarker for human cancer. Although the mechanism remains to be further clarified, an increase in blood viscosity might be associated with the feasibility of circulating tumor cells adhering to the vascular structure, which is linked to distant metastases [17, 18]. However, the clinical effects of the hemorheological alterations in patients with HCC remain unclear.

In the present study, the possible role of WBV as a biomarker in patients with HCC was investigated. Using a historical cohort of treatment-naïve HCC patients, the association between WBV and the metastasis and prognosis in HCC was investigated. In addition, WBV was tested as a predictive marker in patients with low AFP and PIVKA-II levels.

## Materials and methods

### Study patients

This study was approved by the Institutional Review Board (IRB) of the Catholic University of Korea (IRB approval number: KC21RASI0261). Due to the retrospective nature of this study, the informed consent was waived by IRB of the Catholic University of Korea. This study was performed in accordance with the Declaration of Helsinki. Among the historical HCC cohort at Seoul St. Mary’s Hospital, patients who were newly diagnosed with HCC (discovery cohort) or patients who treated with anti-programmed cell death protein-1 (PD-1) nivolumab (validation cohort) between January 2016 and December 2020 were included in this study. The diagnosis of HCC was based on histologic or radiologic examinations such as liver dynamic computed tomography and/or liver dynamic magnetic resonance imaging. Among subjects, patients with preserved liver function were included in the study because decreased liver function, as well as the decompensation, were associated with significant reduction of WBV [11]. Consequently, a total of 148 treatment-naïve HCC patients with preserved liver function who underwent WBV examinations were included for subsequent analyses. In addition, 33 patients who treated nivolumab were also included as a validation cohort. Demographic information, laboratory and radiologic findings at diagnosis of HCC and at the start of nivolumab were recorded, and patients were followed up to the date of death, or the date of last follow-up. Liver stiffness was measured by Fibroscan^®^. Nivolumab (Opdivo^®^) was treated every two weeks, with a dose of 3mg/kg [19]. Tumor response was evaluated every 2–3 months following treatment according to the mRECIST criteria [20].

### Definitions

The degree of portal vein tumor thrombosis (PVTT) was divided into Vp1 to Vp4, as previously described [21]. Preserved liver function was defined as Child-Pugh score ≤ 7 because this cut-off of Child-Pugh scores were shown associated with favorable overall survival (OS) in patients who underwent locoregional [22] or systemic treatments [23]. The HCC stage in the present study was determined using the modified Union for International Cancer Control (mUICC) stage as previously described [20]. When defining AFP/PIVKA-II^lo^ patients, the cut-off point of 20 ng/mL was used for AFP and 40 mAU/mL for PIVKA-II as previously described [24]. Those with both AFP and PIVKA below the cut-off point were defined as the AFP/PIVKA-II^lo^ group. A previous study suggested that 10.3 kPa measured by Fibroscan^®^ predicted the F4 cirrhosis examined by histologic exams [25]. Therefore, we defined LC patients in this study as liver stiffness ≧ 10.3 in the present study.

### WBV

WBV was measured using an automated scanning capillary tube viscometer (Hemovister, South Korea) as previously described [26], which uses whole blood and the gravity-driven disposable U-tube systems. Briefly, 3mL of whole blood collected in an EDTA tube, before the HCC treatment in treatment-naïve patients or before the nivolumab treatment within 3 hours after admission. Because WBV is significantly affected by hemodynamic status, including fluid velocity, WBV was measured at both low shear rate (5 s^-1^), which reflects diastolic WBV, and high shear rate (300 s^-1^), which reflects systolic WBV, and presented as cP.

### Statistical analyses

Graph-Pad Prism version 6.0 (GraphPad Software, USA) and SPSS version 19.0 (IBM Corp., USA) were used for subsequent statistical analyses. Categorical data were presented as the number (%) and continuous data as mean ± standard deviation (SD). Spearman’s rank correlation test was used to measure the correlation between two variables. Mann-Whitney U test was used to compare continuous variables between two unpaired groups. To set the cut-off point for the diastolic WBV, which defines metastasis and non-metastasis groups at the diagnosis of HCC, area under the receiver operating characteristic (AUROC) curve and the Youden index were used. To compare the OS between the low and high diastolic WBV groups, the log-rank test was used. Fisher’s exact test was used to compare categorical variables between the two groups. Univariate and multivariate logistic regression analyses were used to determine the factors associated with extrahepatic metastases, and odds ratio (OR) and confidence interval (CI) were also calculated. Univariate and multivariate Cox regression analyses were used to determine the factors associated with the OS, and hazard ratio (HR) and CI were also calculated. For multivariate logistic and Cox regression analyses, factors with P-values < 0.10 in univariate analyses were included. P-values < 0.05 were considered statistically significant.

## Results

### Correlation between cancer stage and WBV in treatment-naïve HCC

**Fig 1:**
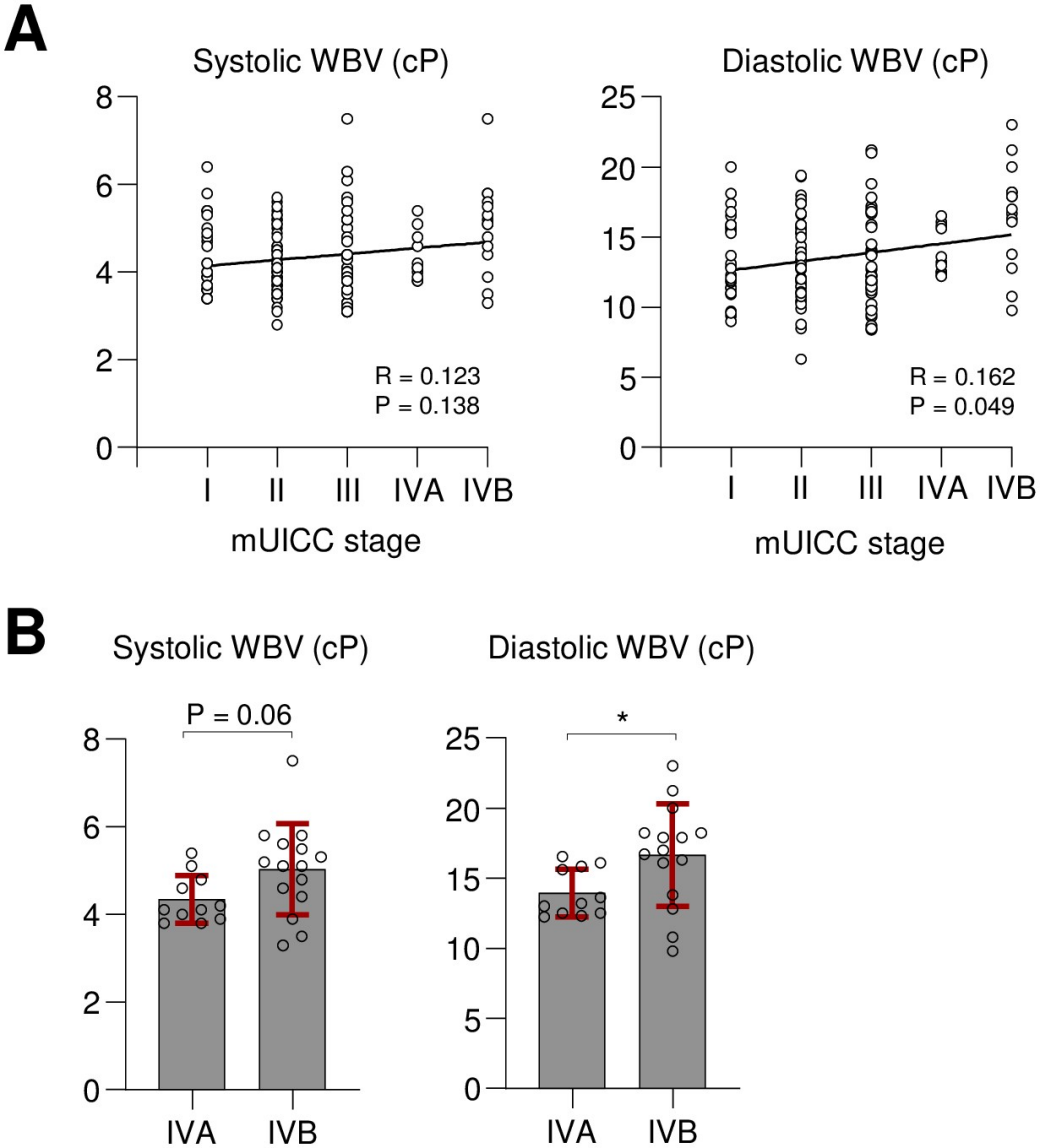
(A) Correlation between systolic WBV (left) or diastolic WBV (right) and HCC stage (mUICC stage) (n = 148) based on Spearman’s rank correlation test. (B) Comparison of systolic WBV (left) or diastolic WBV (right) between patients with stage IVA (n = 11) and stage IVB (n = 15) using Mann-Whitney U test. *P < 0.05.

In a previous study, blood viscosity was reportedly higher in patients with more advanced stage in human gynecologic cancer [15]. Therefore, whether this correlation is also observed in patients with HCC was investigated in the present study. The 148 treatment-naïve HCC patients were divided into five stages, I to IVB, according to the mUICC stage (Fig 1A). Consequently, systolic WBV and mUICC stage were not significantly correlated (P = 0.138, R = 0.123); however, diastolic WBV was positively correlated with mUICC stage (P = 0.049, R = 0.162; Fig 1A). Next, because high blood viscosity was shown associated with cancer metastases in melanomas [16, 27], WBV in two advanced stages of HCC, IVA (n = 11) and IVB (n = 15), were compared in the present study (Fig 1B). Consequently, diastolic WBV was significantly higher in stage IVB (mean 16.7 vs. 13.9 cP, P = 0.013; Fig 1B). These results suggest that high WBV is also associated with advanced stage of treatment-naïve HCC, which might be associated with extrahepatic metastases.

### Baseline characteristics of patients with or without extrahepatic metastases

Next, the 148 patients were divided into two groups, patients with metastases (metastases group, n = 15) and without metastases (non-metastases group, n = 133) and the baseline characteristics were compared (Table 1). The median follow-up time was 1,110 days. Age, sex, and HCC etiology were not different between the two groups. Histories of diabetes mellitus and dyslipidemia, and the uses of antiplatelet agents including low-dose aspirin or clopidogrel, and anticoagulants including warfarin or new oral anticoagulants were also not significantly different between the metastases and non-metastases groups. In addition, liver function such as total bilirubin, albumin, and international normalized ratio (INR), as well as Child-Pugh class, were not different between the two groups. Tumor markers such as AFP and PIVKA-II were also similar in the two groups. However, the frequency of patients with PVTT was significantly higher in the metastases group than in the non-metastases group (7/15, 46.7% vs. 18/133, 13.5%, P = 0.004), and advanced PVTT (Vp3 and Vp4) was also more frequent in the metastases group (3/15, 20% vs. 6/133, 4.5%). Hepatic venous tumor thrombosis was also tended to be frequent in the metastases group (2/15, 13.3% vs. 2/133, 1.5%). These findings suggest that the metastases group also has more advanced vascular involvement than the non-metastases group. Largest size and number of intrahepatic tumors were also significantly higher in the metastases group (P < 0.001). Furthermore, the number of patients with multiple tumors tended to be higher in the metastases group (10/15, 66.6% vs. 49/133, 36.8%, P = 0.058). These findings indicate that patients with extrahepatic metastases have higher intrahepatic tumor burden. Furthermore, more patients with metastases died during the follow-up period (13/15, 86.7% vs. 37/133, 27.8%, P < 0.001) and the major cause of death was HCC progression (11/13, 84.6%). The proportion of LC between non-metastases and metastases groups was not significantly different (66/133, 49.6% versus 9/15, 60.0%, P = 0.588).

**Table 1:** Baseline characteristics of HCC patients with extrahepatic metastases and without metastasis.

	Non-metastases	Metastases	p
	(n = 133)	(n = 15)	
Age, years	64.1 ± 11.9	61.5 ± 8.0	0.408
Male gender	103 (77.4)	11 (73.3)	0.972
Underlying liver disease			0.695
HBV	74 (55.6)	10 (66.7)	
HCV	17 (12.8)	0 (0.0)	
Alcohol	17 (12.8)	2 (13.3)	
NASH	17 (12.8)	2 (13.3)	
Others	8 (6.0)	1 (6.7)	
Diabetes mellitus	39 (29.3)	3 (20.0)	0.648
Dyslipidemia	24 (18.0)	0 (0.0)	0.153
Antiplatelet	23 (17.3)	0 (0.0)	0.169
Anticoagulant	5 (3.8)	0 (0.0)	0.992
TB, mg/dL	1.1 ± 1.0	0.9 ± 0.5	0.195
Albumin, g/dL	3.8 ± 0.5	3.7 ± 0.4	0.293
INR	1.2 ± 0.3	1.2 ± 0.2	0.893
Liver stiffness, kPa	17.2 ± 15.8	20.3 ± 16.4	0.373
Liver cirrhosis	66 (49.6)	9 (60.0)	0.588
Child-Pugh Class			0.563
A	116 (87.2)	12 (80.0)	
B	17 (12.8)	3 (20.0)	
AFP, ng/mL	8971.0 ± 70310.9	8580.1 ± 31637.8	0.970
PIVKA-II, mAU	9326.7 ± 37294.1	19010.7 ±31817.6	0.336
PVTT	18 (13.5)	7 (46.7)	***0*.*004***
Vp1	1 (0.8)	1 (6.7)	
Vp2	3 (2.3)	1 (6.7)	
Vp3	7 (5.3)	2 (13.3)	
Vp4	6 (4.5)	3 (20.0)	
Hepatic vein tumor thrombosis	2 (1.5)	2 (13.3)	0.066
Stage (mUICC)			***< 0*.*001***
I	28 (21.1)	0 (0.0)	
II	53 (39.8)	0 (0.0)	
III	41 (30.8)	0 (0.0)	
IVA	11 (8.3)	0 (0.0)	
IVB	0 (0.0)	15 (100.0)	
Largest tumor size, cm	5.1 ± 4.7	11.5 ± 5.1	***< 0*.*001***
Total tumor size, cm	5.9 ± 5.5	13.7 ± 5.4	***< 0*.*001***
Tumor number			0.058
Single	84 (63.2)	5 (33.3)	
Multiple	49 (36.8)	10 (66.6)	
Death	37 (27.8)	13 (86.7)	***< 0*.*001***
Cause of death			***< 0*.*001***
HCC progression	22 (59.5)	11 (84.6)	
Hepatic failure	10 (27.0)	1 (7.7)	
Infection	15 (40.5)	1 (7.7)	
Systoloic WBV, cP	4.3 ± 0.8	5.0 ± 1.0	***0*.*001***
Diastolic WBV, cP	13.3 ± 3.0	16.6 ± 3.6	***< 0*.*001***

Data are given as n (%) or mean ± SD. HCC, hepatocellular carcinoma; HBV, hepatitis B virus; HCV, hepatitis C virus; NASH, non-alcoholic steatohepatitis; TB, total bilirubin; INR, international normalized ratio; AFP, alpha-fetoprotein; PIVKA-II, proteins induced by vitamin K antagonist-II; PVTT, portal vein tumor thrombosis; mUICC, modified international union against cancer; WBV, whole blood viscosity.

### Association of high diastolic WBV and extrahepatic metastases

**Fig 2:**
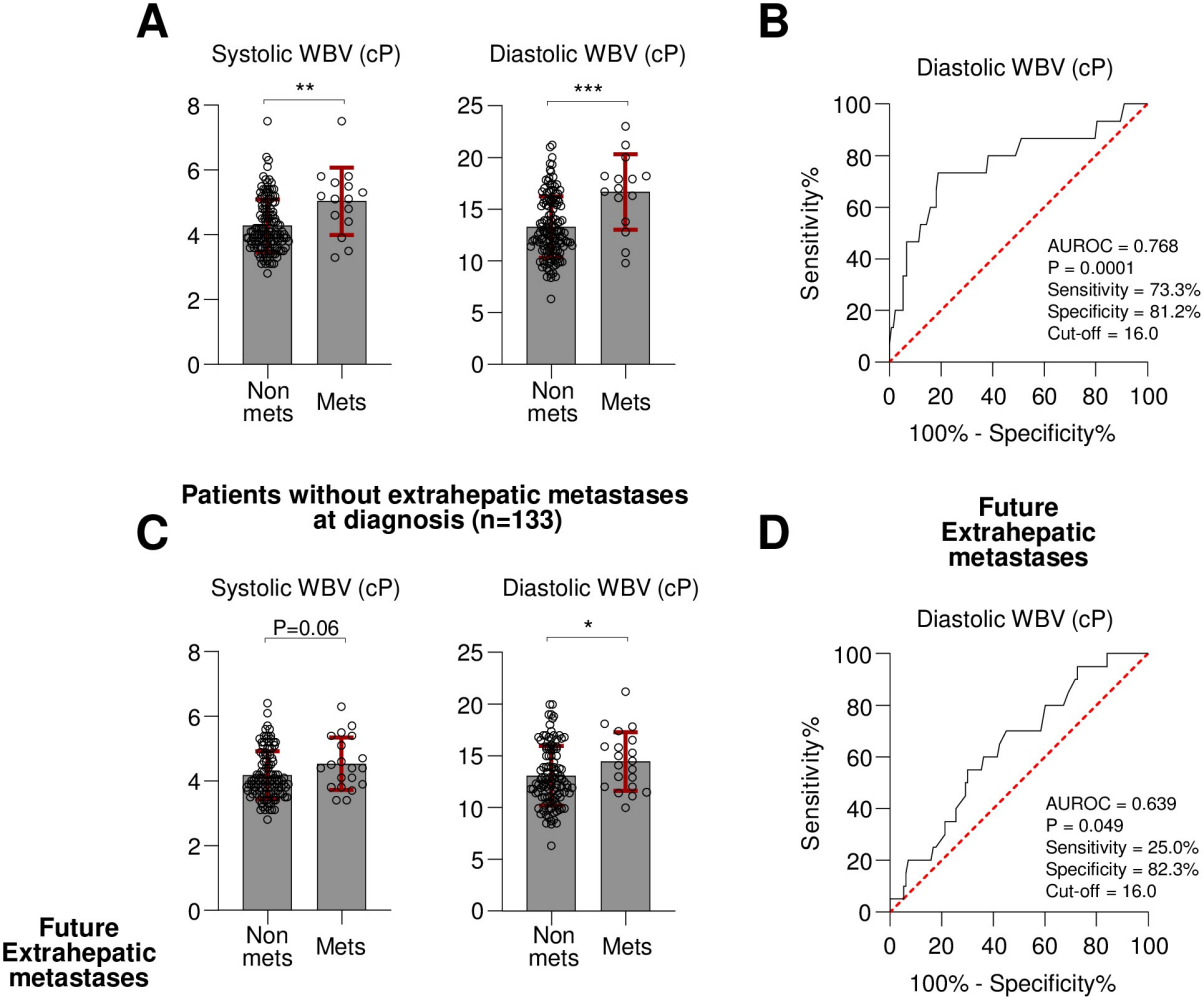
(A and B) All 148 enrolled patients were evaluated for subsequent analyses. (A) Comparison of systolic WBV (left) or diastolic WBV (right) between patients with non-metastases (non-mets) (n = 133) and metastases (mets) (n = 15) based on Mann-Whitney U test. (B) AUROC curve analysis was performed to determine the power of diastolic WBV in predicting metastases at diagnosis of HCC. (C and D) The 133 patients without metastases at diagnosis were evaluated for subsequent analyses. Future metastases were diagnosed during the follow-up periods in 20 patients. (C) Comparison of systolic WBV (left) or diastolic WBV (right) between patients with future non-metastases (non-mets) (n = 113) and future metastases (mets) (n = 20) based on Mann-Whitney U test. (D) AUROC curve analysis was performed to determine the power of diastolic WBV in predicting future metastases. *P < 0.05, **P < 0.01, ***P < 0.001.

Next, systolic and diastolic WBV were compared in the metastases and non-metastases groups. Consequently, systolic WBV was significantly higher in the metastases group (mean 5.0 vs. 4.3 cP, P = 0.001; Table 1 and Fig 2A). In addition, diastolic WBV was higher in the metastases group (mean 16.6 vs. 13.3 cP, P < 0.001; Table 1 and Fig 2A). Due to the subtle difference of systolic WBV, diastolic WBV was used to set a cut-off point for dividing the patients into high and low WBV groups. The AUROC curve analysis showed diastolic WBV significantly predicted extrahepatic metastases (AUROC = 0.768, P < 0.001) with an optimal cut-off point of 16.0 cP (Fig 2B). To determine the independent factors associated with extrahepatic metastases, logistic regression analyses were performed (Table 2). PVTT, largest size and number of intrahepatic tumors, and high diastolic WBV were significant factors in univariate analysis. However, high diastolic WBV only remained a significant factor in multivariate analysis (OR = 23.41, P < 0.001). These results suggest that high diastolic WBV is an independent factor associated with extrahepatic metastases in treatment-naïve HCC patients.

**Table 2:** Univariate and multivariate logistic regression analysis for factors associated with the extrahepatic metastases.

	Univariate		Multivariate	
	OR (95% CI)	p	OR (95% CI)	p
PVTT	5.56 (1.75–16.67)	.003		.369
Largest tumor size	1.22 (1.11–1.34)	< .001		.656
Total tumor size	1.20 (1.11–1.32)	< .001		.064
Tumor number	1.58 (1.14–2.20)	.006		.942
High diastolic WBV (> 16.0)	11.88 (3.73–45.77)	< .001	23.41 (5.27–157.76)	***<* .*001***

OR, odds ratio; CI, confidence interval; PVTT, portal vein tumor thrombosis; WBV, whole blood viscosity.

Next, whether the initial high WBV can predict future extrahepatic metastases in patients without metastases (n = 133) was analyzed. First, systolic WBV differences between patients with (n = 20) and without (n = 113) future metastases were compared; the future metastases group tended to have higher systolic WBV (mean 4.5 vs. 4.1 cP, P = 0.058) (Fig 2C). Furthermore, the future metastases group had significantly higher diastolic WBV than the non-metastases group (mean 14.5 vs. 13.0 cP, P = 0.048; Fig 2C). In the AUROC curve analysis, diastolic WBV could predict future extrahepatic metastases (AUROC = 0.639, P = 0.049; Fig 2D). When we compared the other characteristics between future non-metastases and future metastases groups among patients with the initial non-metastases, the largest tumor size (mean 11.5cm versus 5.1cm, P = 0.007) and PVTT (30.0% versus 10.6%, P = 0.048) were also different factors (S1 Table). In Cox regression analyses, PVTT and the largest tumor size were significant factors both in the univariate and multivariate analyses (S2 Table). On the other hand, high diastolic WBV had a weak association in univariate (P = 0.06) and multivariate (P = 0.10) analyses (S2 Table).

### Comparison of the characteristics between the high and low diastolic WBV groups

Next, the characteristics in high (n = 36) and low (n = 112) diastolic WBV groups were compared (Table 3). Systolic WBV (mean 5.4 vs. 4.0 cP, P < 0.001) and diastolic WBV (mean 18.0 vs. 12.2, P < 0.001) were higher in the high diastolic WBV group. Liver function, liver stiffness, tumor markers, intrahepatic tumor burden, and PVTT did not differ between the two groups. We also compared the degree of PVTT between the high and low WBV groups but found that advanced PVTT (Vp3 and Vp4) was not significantly different between the two groups (6/36, 16.7% vs. 12/112, 10.7%, P = 0.224). Hepatic vein tumor thrombosis was also not different between the two groups. Additionally, because high WBV is a risk factor for the type 2 DM [28] and dyslipidemia is associated with the high WBV [29], we compared these metabolic factors between high and low diastolic WBV groups, but there was no significant difference (Table 3). When we compared the uses of antiplatelet agents and anticoagulants between high and low WBV groups, there was no significant difference. Hematocrit (P < 0.001) and total protein level (P = 0.046) were higher in the high diastolic WBV group. Because hematocrit and total protein level can affect WBV, we evaluated the ratio of diastolic WBV to hematocrit and total protein (S1 Fig), but the ratios were also significantly higher in the high diastolic WBV group, indicating WBV might not be affected by them alone.

**Table 3:** Comparison between the patients with high (> 16.0 cP) diastolic WBV and low diastolic WBV (≦16.0 cP) group.

	Low diastolic WBV	High diastolic WBV	p-value
	(n = 112)	(n = 36)	
Systoloic WBV, cP	4.0 ± 0.6	5.4 ± 0.7	***< 0*.*001***
Diastolic WBV, cP	12.2 ± 2.1	18.0 ± 1.7	***< 0*.*001***
Age, years	64.3 ± 11.6	62.3 ± 11.4	0.369
Male gender	83 (74.1)	31 (86.1)	0.207
Underlying liver disease			0.521
HBV	63 (56.2)	21 (58.3)	
HCV	15 (13.4)	2 (5.6)	
Alcohol	12 (10.7)	7 (19.4)	
NASH	15 (13.4)	4 (11.1)	
Others	7 (6.2)	2 (5.6)	
Diabetes mellitus	33 (29.5)	9 (25.0)	0.761
Dyslipidemia	22 (19.6)	2 (5.6)	0.083
Antiplatelet	19 (17.0)	4 (11.1)	0.563
Anticoagulant	5 (4.5)	0 (0.0)	0.448
TB, mg/dL	1.0 ± 1.0	1.1 ± 1.1	0.580
Albumin, g/dL	3.8 ± 0.6	3.9 ± 0.5	0.399
INR	1.2 ± 0.3	1.2 ± 0.2	0.619
Hematocrit, %	38.5 ± 5.1	43.0 ± 5.2	***< 0*.*001***
Total protein, g/dL	6.9 ± 0.6	7.2 ± 0.5	***0*.*046***
Liver stiffness, kPa	16.6 ± 15.6	19.8 ± 16.4	0.125
Child-Pugh Class			0.698
A	96 (85.7)	32 (88.9)	
B	16 (14.3)	4 (11.1)	
AFP, ng/mL	8887.7 ± 75818.5	9067.4 ± 28533.7	0.983
PIVKA, mAU	11303.3 ± 40696.2	7212.4 ± 20519.9	0.428
PVTT	17 (15.2)	8 (22.2)	0.468
Vp1	1 (0.9)	1 (2.8)	
Vp2	3 (2.7)	1 (2.8)	
Vp3	7 (6.2)	2 (5.6)	
Vp4	5 (4.5)	4 (11.1)	
Hepatic vein tumor thrombosis	2 (1.8)	2 (5.6)	0.534
Stage (mUICC)			***< 0*.*001***
I	23 (20.5)	5 (13.9)	
II	44 (39.3)	9 (25.0)	
III	32 (28.6)	9 (25.0)	
IVA	9 (8.0)	2 (5.6)	
IVB	4 (3.6)	11 (30.6)	
Extrahepatic metastases	4 (3.6)	11 (30.6)	***< 0*.*001***
Lung	2 (50.0)	9 (81.8)	
Lymph nodes	1 (25.0)	0 (0.0)	
Adrenal gland	0 (0.0)	1 (9.1)	
Bone	2 (50.0)	3 (27.3)	
Largest tumor size, cm	5.5 ± 4.9	6.6 ± 5.7	0.255
Total tumor size, cm	6.4 ± 5.9	7.6 ± 6.1	0.276
Tumor number			0.102
Single	71 (63.4)	18 (50.0)	
Multiple	41 (36.6)	18 (50.0)	
Death	27 (24.1)	23 (63.9)	***< 0*.*001***
Cause of death			***< 0*.*001***
HCC progression	13 (48.1)	20 (87.0)	
Hepatic failure	10 (37.0)	1 (4.3)	
Infection	4 (14.8)	2 (8.7)	

Data are given as n (%) or mean ± SD. WBV, whole blood viscosity. HBV, hepatitis B virus; HCV, hepatitis C virus; NASH, non-alcoholic steatohepatitis; TB, total bilirubin; INR, international normalized ratio; AFP, alpha-fetoprotein; PIVKA-II, proteins induced by vitamin K antagonist-II; PVTT, portal vein tumor thrombosis; mUICC, modified international union against cancer.

Interestingly, the frequency of extrahepatic metastases was significantly higher in the high diastolic WBV group (11/36, 30.6% vs. 4/115, 3.6%, P < 0.001). Furthermore, more patients died in the high diastolic WBV group than in the low diastolic WBV group during the observation periods (23/36, 63.9% vs. 27/115, 24.1%, P < 0.001). The major cause of death in the high diastolic WBV group was HCC progression (20/23, 87.0%). These findings indicated that high diastolic WBV might be associated with extrahepatic metastases and poor survival.

### High diastolic WBV as a poor prognostic factor in HCC patients

**Fig 3:**
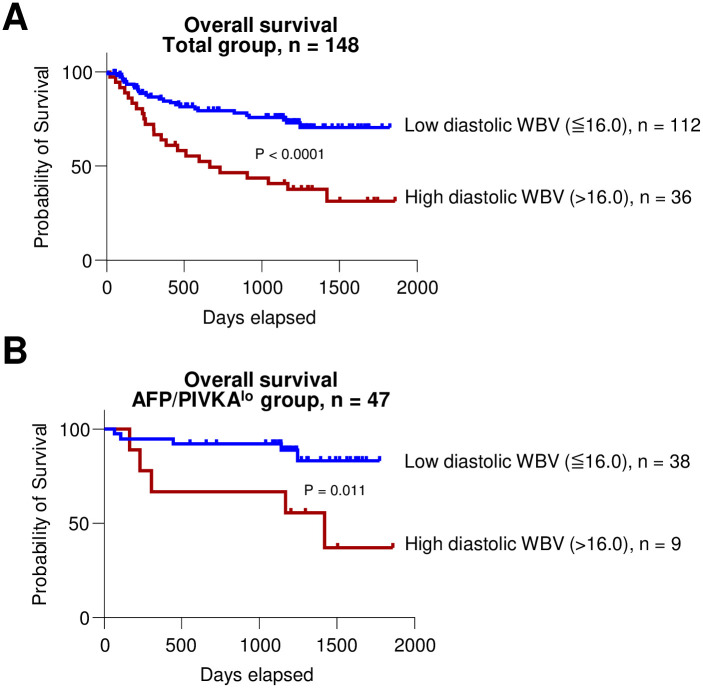
(A and B) Kaplan-Meier curves for comparing OS between low diastolic WBV (≤ 16.0) and high diastolic WBV (> 16.0) groups in all patients (n = 148) (A) and in patients with low AFP/PIVKA (AFP < 20 ng/mL and PIVKA < 40 mAU/mL) (n = 47) (B).

Finally, survival analyses were performed to determine whether high diastolic WBV can be a prognostic factor in treatment-naïve HCC patients. First, a log-rank test was performed in all groups; significant difference in survival was observed between high (n = 36) and low (n = 112) groups (P < 0.001; Fig 3A). Next, Cox regression analyses were performed to investigate the factors associated with poor survival (Table 4). Consequently, Child-Pugh B7, tumor size, and high diastolic WBV, were significant factors in univariate analysis. However, in multivariate analysis, Child-Pugh B7 (HR 2.99, P < 0.001), PVTT (HR 3.16, P < 0.001), and high diastolic WBV (HR 3.81, P < 0.001), were significant factors associated with poor survival. Although the overall staging was not a statistically significant factor for poor survival, the advanced stage was significant only in the univariate analysis (HR = 2.23, P = 0.003), but not in the multivariate analysis (P = 0.06). Notably, in the subgroup analysis of patients with low AFP/PIVKA (n = 47) at diagnosis, difference in survival measured using the log-rank test between high (n = 9) and low (n = 38) diastolic WBV groups was also observed (P = 0.011; Fig 3B). Taken together, these findings indicated that high diastolic WBV is an independent risk factor associated with poor survival in treatment-naïve HCC patients, which can also be applicable in patients with low AFP/PIVKA.

**Table 4:** Univariate and multivariate Cox regression analysis for factors associated with the overall survival.

	Univariate		Multivariate	
	HR (95% CI)	p	HR (95% CI)	p
Child-Pugh class B	2.40 (1.34–4.29)	.003	2.99 (1.65–5.41)	***<* .*001***
Stage	1.20 (0.95–1.52)	.118		.116
Advanced stage (IVA / IVB)	2.23 (1.08–4.89)	.003	1.81 (1.01–1.75)	.061
PVTT	2.70 (1.37–5.26)	.004	3.16 (1.43–7.04)	***<* .*001***
Large tumor size	1.04 (1.00–1.08)	.070		.731
Total tumor size	1.04 (1.00–1.08)	.035		.691
High diastolic WBV (> 16.0)	2.57 (1.39–4.74)	.003	3.81 (1.98–7.35)	***<* .*001***

OR, odds ratio; CI, confidence interval; PVTT, portal vein tumor thrombosis; WBV, whole blood viscosity.

**Fig 4:**
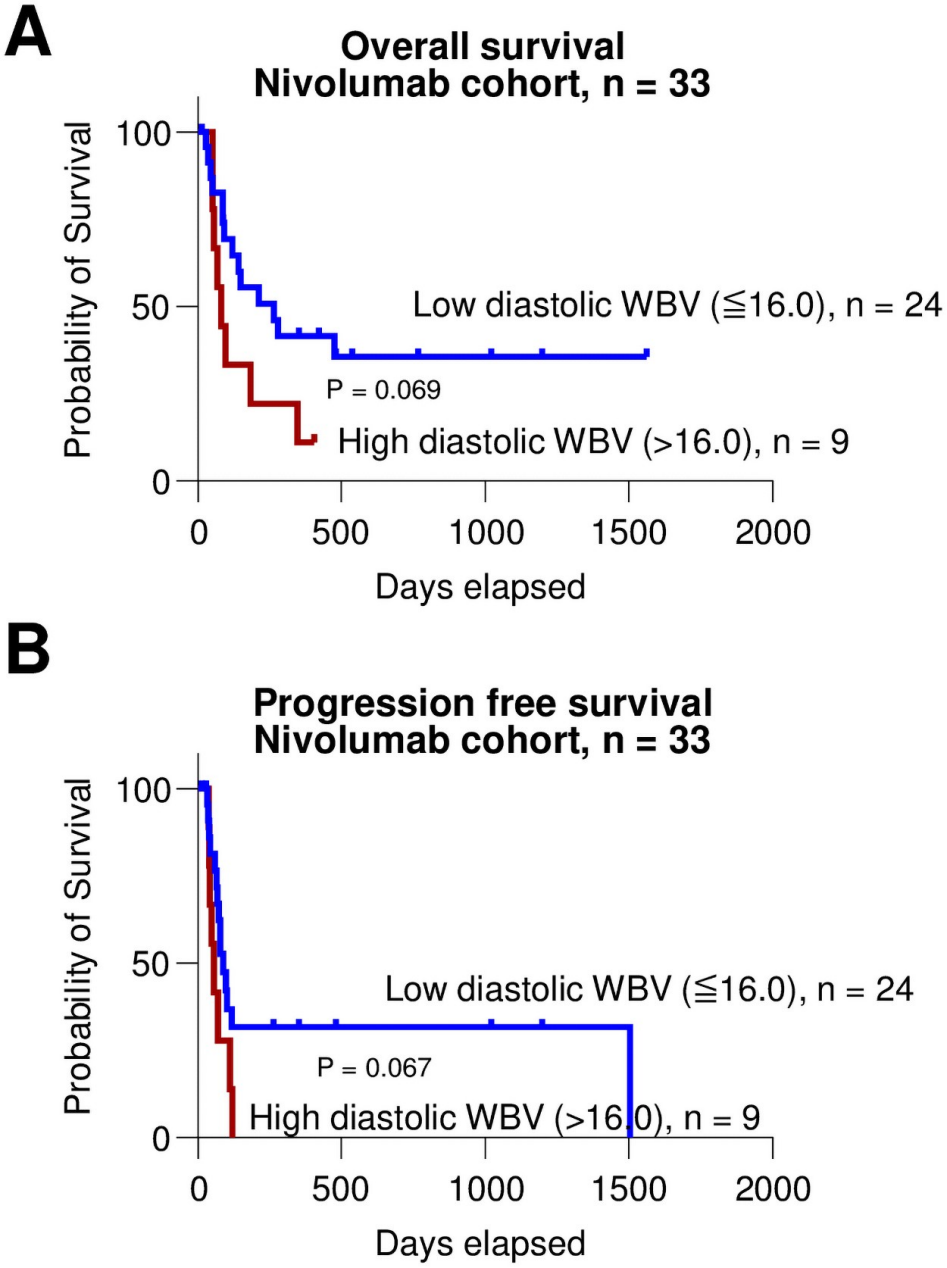
(A and B) Kaplan-Meier curves for comparing OS (A) and PFS (B) between low diastolic WBV (≤ 16.0, n = 24) and high diastolic WBV (> 16.0, n = 9) groups in nivolumab cohort (n = 33).

Finally, we validated whether WBV can be a predictive marker for outcomes of patients who treated with nivolumab (n = 33). Comparison of characteristics between low (n = 24) and high (n = 9) group is presented in Table 5. All patients had a previous treatment history for HCC. There was no significant difference in the Child-Pugh class and laboratory tests. Most patients had extrahepatic metastases, both in the low (19/24, 79.2%) and high (8/9, 88.9%) group, without significant difference. In terms of the best response, there were 6 responders among low group (1 of complete response, 1/24, 4.2% and 5 of partial response, 5/24, 20.8%), but there was no responder in the high group (P = 0.409). During the median 143 days of observation period, 14 (58.3%) patients among low group and 8 (88.9%) patients among high group died due to the cancer progression, without significant significance (P = 0.137) which might be due to the small sample size. When we compared the OS between two group (Fig 4A), low group showed a tendency of better OS than high group (P = 0.069). In terms of progression free survival (PFS) (Fig 4B), low group showed a tendency of longer PFS than high group (P = 0.067).

**Table 5:** Comparison between the patients with high (> 16.0 cP) diastolic WBV and low diastolic WBV (≦16.0 cP) patients with nivolumab treatment.

	Low diastolic WBV	High diastolic WBV	p-value
	(n = 24)	(n = 9)	
Systoloic WBV, cP	3.8 ± 0.4	5.1 ± 0.4	***< 0*.*001***
Diastolic WBV, cP	11.7 ± 1.5	17.3 ± 1.1	***< 0*.*001***
Age, years	60.8 ± 11.1	56.3 ± 9.3	0.294
Male gender	19 (79.2)	7 (77.8)	1.000
Underlying liver disease			0.553
HBV	19 (79.2)	8 (88.9)	
HCV	2 (8.3)	0 (0.0)	
Alcohol	2 (8.3)	0 (0.0)	
Other	1 (4.2)	1 (11.1)	
TB, mg/dL	1.0 ± 0.7	1.1 ± 0.5	0.843
Albumin, g/dL	3.6 ± 0.6	3.6 ± 0.4	0.888
INR	1.1 ± 0.1	1.2 ± 0.2	0.293
Child-Pugh Class			0.249
A	18 (75.0)	9 (100.0)	
B	6 (25.0)	0 (0.0)	
AFP, ng/mL	17096.3 ± 44634.5	10829.4 ± 19856.1	0.582
PIVKA, mAU	8138.4 ± 11776.0	20152.6 ± 40480.8	0.405
Previous treatment history	23 (100.0)	9 (100.0)	1.000
Extrahepatic metastases	19 (79.2)	8 (88.9)	0.890
Stage (mUICC)			0.658
I	0 (0.0)	0 (0.0)	
II	2 (8.3)	0 (0.0)	
III	3 (12.5)	1 (11.1)	
IVA	0 (0.0)	0 (0.0)	
IVB	19 (79.2)	8 (88.9)	
Best response			0.409
CR	1 (4.2)	0 (0.0)	
PR	5 (20.8)	0 (0.0)	
SD	3 (12.5)	2 (22.2)	
PD	15 (62.5)	7 (77.8)	
Death	14 (58.3)	8 (88.9)	0.137

Data are given as n (%) or mean ± SD. WBV, whole blood viscosity. HBV, hepatitis B virus; HCV, hepatitis C virus; TB, total bilirubin; INR, international normalized ratio; AFP, alpha-fetoprotein; PIVKA-II, proteins induced by vitamin K antagonist-II; mUICC, modified international union against cancer; BCLC, Barcelona Clinic Liver Cancer; CR, complete response; PR, partial response; SD, stable disease; PD, progressive disease.

## Discussion

In the present study, we firstly evaluated the clinical relevance of blood viscosity in patients with HCC. The correlation of blood viscosity level and HCC stage, as previously reported in other cancers, was confirmed, which might be associated with distant metastases. Patients with distant, extrahepatic metastases had significantly higher systolic and diastolic WBV, and diastolic WBV was an independent associated factor. Notably, higher diastolic WBV was also associated with the future development of metastases, indicating a close initial and subsequent evaluation for identifying metastases should be performed in patients with high blood viscosity. Notably, high diastolic WBV was an independent factor for predicting poor survival, and this result was also observed in AFP/PIVKA^lo^ patients. Furthermore, we validated these results in patients received nivolumab in terms of OS and PFS, indicating that blood viscosity could be used as a novel biomarker in HCC patients, although future large-scale prospective studies are necessary to validate our results.

There are numerous factors affecting cancer metastases. Recent research has concentrated on cellular and molecular factors, which is based on “seed and soil” theory [30]. For example, circulating tumor cells (CTCs) can be affected by intrinsic mechanisms such as epithelial-to-mesenchymal transition, stemness, metastatic dormancy, or autophagy, and can be affected by extrinsic mechanisms such as tumor-secreted chemokines or cytokines, and extracellular vesicles, resulting in the enhancement of metastatic potential of the CTCs [31]. Furthermore, the tumor microenvironment, such as hypoxia, cellular components including macrophages, mesenchymal stem cells, endothelial cells, and fibroblasts, have a critical role in regulating cancer metastases [32]. However, mechanical factors, including the structure of distant organs and hemodynamic changes of the vascular system, can also affect cancer metastases (hemodynamic theory) [18]. The importance of the vascular network in cancer metastases was confirmed in several previous reports [33–36]. In venous flow with low velocity, survival, arrest, and colonization of CTCs are relatively feasible, and the major determinant of fluid dynamics is viscosity [37, 38]. These theories were also confirmed in the experimental mouse hepatoma model of distant metastases, which showed that blood viscosity was significantly increased in the early stage of metastases [39].

Based on the evidence, the association of metastases and clinical outcome with blood viscosity in several types of cancer was investigated in previous studies. In patients with head and neck cancers, significantly high WBV was observed in stage IV patients with distant metastases [13]. Melanoma patients with distant metastases had significantly elevated blood viscosity compared with patients without metastases [16, 27]. However, the correlation between metastases and blood viscosity in HCC patients has not been previously studied. To the best of our knowledge, this is the first study in which the association between metastases and WBV was clearly shown in the historical cohort of treatment-naïve HCC patients using a new scanning tube viscometer that is a relatively fast and precise method. Notably, the data showed diastolic WBV, rather than systolic WBV, had more clinical relevance in terms of the association with metastases and survival, which is consistent with previous reports that blood viscosity is more important when the flow velocity is low [37, 38].

Since the metastasis of HCC involves not only venous implantation but also trans-portal dissemination, the role of adhesion factors should be considered in relation to WBV. Adhesion factors such as PECAM-1 and VE-cadherin are known to be key factors of flow mechanosensing and might be closely related to the blood viscosity [40]. Both VE-cadherin and PECAM-1-based adhesions induce integrin activation by the fluid shear stress [41]. Because VE-cadherin [42] and PECAM-1 [43] expressed in the HCC cells, there is a possibility that increased blood viscosity can affect the metastases via trans-portal dissemination via the alteration of adhesion molecules. Importantly, PECAM-1 [43] and VE-cadherin [44] regulate HCC metastasis via epithelial-mesenchymal transition. Therefore, both mechanical and molecular mechanisms associated with WBV might be involved in HCC metastasis, as well as cancer progression, although future studies are needed.

Several biomarkers which can be used in tumor staging and predicting survival have been previously suggested. AFP is a representative predictive marker of prognosis and has been widely used in the diagnosis of HCC. In the early [45, 46] and advanced stages [47], AFP is a significant risk factor for poor patient survival. Furthermore, AFP-L3 has been studied as a prognostic factor. High AFP-L3 [48] and PIVKA-II [49] levels were also associated with poor survival. However, in a previous study, 23.2% of HCC patients did not have these three representative tumor markers [3], showing the need for developing other supportive biomarkers. For example, upregulation of osteopontin was associated with development of metastasis and poor survival in surgically resected HCC [50]. In addition, micro-RNAs have been shown a predictive factor for metastasis and poor survival in HCC patients [51, 52]. Similarly, the possibility of using blood viscosity as a novel biomarker was shown in the present study, in treatment-naïve and nivolumab-treated patients. Notably, it might be one of the promising biomarker in patients receiving immunotherapy, because there is no reliable biomarker for the immunotherapy in HCC. Furthermore, it can be examined rapidly and constantly using a new scanning capillary viscometer in a standardized manner using peripheral blood. However, multicenter, large-scale, prospective studies are necessary to validate our results.

Although there was no statistical significance due to the small sample size, the objective response rate was tended to be higher in the low diastolic WBV group compared to the high diastolic WBV group (25.0% versus 0%). Furthermore, the disease control rate was also tended to be higher in the low diastolic WBV group (37.5% versus 22.2%). We could not investigate the various inflammatory markers in this manuscript, but a chronic increase of inflammatory mediators and its association with poor survival in patients with HCC has been reported by previous reports [53]. In inflammatory conditions, blood viscosity is increased and correlated with acute-phase proteins [54]. The interaction of systemic inflammation and blood viscosity improved the prediction of cardiovascular disease in a previous report [55]. On the other hand, systemic inflammation can limit tumor-specific immunity and responses to the immune-checkpoint inhibitors (ICIs). In a recent study, systemic inflammation demonstrated by increased cytokine profiles was associated with poor survival and response to the ICIs in patients with lung cancer [56]. Therefore, blood viscosity can also be a biomarker for HCC patients treated with ICIs, and future large and prospective study is needed.

In previous studies, low-dose aspirin was suggested to reduce cancer metastasis and improve patient survival in various types of cancer [57–59]. Several mechanisms of aspirin-inhibited metastasis have been suggested, such as the regulation of angiogenesis [60], modulation of the NF-κB pathway [61], or inhibition of toll-like receptor 4 [62]. Furthermore, aspirin can suppress the acquisition of cancer cell chemoresistance [63]. Because increased blood viscosity is associated with cancer metastasis and poor survival, whether reduction in blood viscosity can improve the outcome of cancer patients needs to be elucidated. Low-dose aspirin can significantly reduce WBV in patients with cardiovascular disease [64]; however, the effects of aspirin in reducing blood viscosity and its association with reduction of metastases and improvement of the outcome, especially in HCC patients, requires investigation in future studies. In addition, anticoagulants such as warfarin can significantly reduce WBV [64], and has been suggested to have antitumor and antimetastatic effects [65]. Therefore, these strategies can be applied to HCC patients with high WBV. Notably, in a recent study, low-dose aspirin was shown to reduce HCC development in patients with chronic hepatitis [66]. However, the use of aspirin or anticoagulants should be cautiously considered because patients with impaired liver function have significantly high bleeding risk, and patients with advanced LC appear to have significantly low WBV [4–7].

The present study had several limitations. First, this study used a historical, retrospective cohort. Second, the sample size was relatively small.

In conclusion, diastolic WBV was associated with distant metastases and survival in patients with treatment-naïve and nivolumab-treated HCC patients who have preserved liver function. Although there were some limitations, this is the first study which showed the clinical relevance of blood viscosity in patients with HCC. Future prospective, large-scale studies are necessary to validate our results.

## Supporting information

S1 Fig Comparison of the ratio for diastolic WBV to hematocrit (left) and the ratio for diastolic WBV to total protein (right) in patients with low diastolic WBV (≤ 16.0, n = 112) and high diastolic WBV (> 16.0, n = 36) groups. ****P < 0.0001.
(PDF)

S1 Table Significantly different factors between the patients with future metastases and non-metastases among patients without initial metastases. (PDF)

S2 Table Univariate and multivariate Cox regression analysis for factors associated with the future metastases among patients without initial metastases. (PDF)

## Abbreviations

AFP: alpha-fetoprotein
AUROC: area under the receiver operating characteristic
CI: confidence interval
CTCs: circulating tumor cells
HBV: hepatitis B virus
HCC: hepatocellular carcinoma
HR: hazard ratio
INR: international normalized ratio
IRB: Institutional Review Board
LC: liver cirrhosis
mUICC: modified Union for International Cancer Control
OR: odds ratio
OS: overall survival
PD-1: programmed cell death protein-1
PFS: progression free survival
PIVKA-II: proteins induced by vitamin K antagonist-II
PVTT: Portal vein tumor thrombosis
SD: standard deviation
WBV: Whole blood viscosity
