# Supplementary material for: Whole blood viscosity is associated with extrahepatic metastases and survival in patients with hepatocellular carcinoma
Source: PLoS One. 2021 Dec 2;16(12):e0260311. doi: 10.1371/journal.pone.0260311 (PMC8638904; doi:10.1371/journal.pone.0260311)
Supplement: S1 Fig — ****P < 0.0001. (PDF) [file pone.0260311.s001.pdf]

S1 Fig.

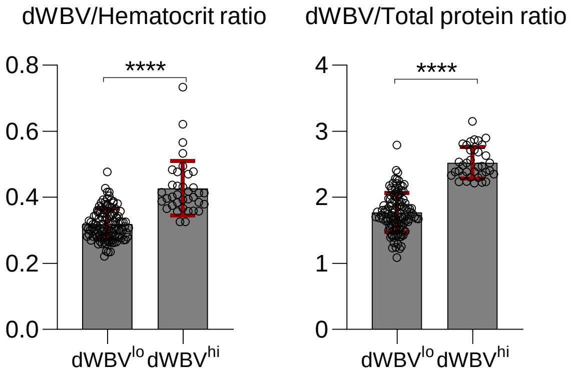

S1 Fig. Comparison of the ratio for diastolic WBV to hematocrit (left) and the ratio for diastolic WBV to total protein (right) in patients with low diastolic WBV (≤ 16.0, n = 112) and high diastolic WBV (> 16.0, n = 36) groups. ****P < 0.0001.
