# Supplementary material for: Whole blood viscosity is associated with extrahepatic metastases and survival in patients with hepatocellular carcinoma
Source: PLoS One. 2021 Dec 2;16(12):e0260311. doi: 10.1371/journal.pone.0260311 (PMC8638904; doi:10.1371/journal.pone.0260311)
Supplement: S1 Table — (PDF) [file pone.0260311.s002.pdf]

S1 Table. Significantly different factors between the patients with future metastases and non-metastases among patients without initial metastases

	Future non-metastases (n=113)	Future metastases (n=20)	p-value
Age, years	60.4 ± 10.3	64.7 ± 12.1	0.128
Male gender	17 (85.0)	86 (76.1)	0.557
Child-Pugh Class			0.594
A	95 (84.0)	20 (100)	
B	18 (16.0)	0 (0.0)	
AFP, ng/mL	47522.5 ± 177857.9	2147.8 ± 12151.8	0.268
PIVKA-II, mAU	28294.0 ± 82364.5	5969.7 ± 20316.4	0.242
PVTT	12 (10.6)	6 (30.0)	***0.048***
Stage (mUICC)			0.120
I	1 (5.0)	27 (23.9)	
II	7 (35.0)	46 (40.7)	
III	9 (45.0)	32 (28.3)	
IVA	3 (15.0)	8 (7.1)	
Largest tumor size, cm	5.1 ± 4.7	11.5 ± 5.1	***0.007***
Tumor number			
Single	71 (62.8)	13 (65.0)	
Multiple	42 (37.2)	7 (35.0)	
Systoloic WBV, cP	4.5 ± 0.8	4.1 ± 0.8	0.058
Diastolic WBV, cP	13.0 ± 2.9	14.4 ± 3.0	***0.048***

Data are given as n (%) or mean ± SD. AFP, alpha-fetoprotein; PIVKA-II, proteins induced by vitamin K antagonist-II; PVTT, portal vein tumor thrombosis; mUICC, modified international union against cancer; WBV, whole blood viscosity.
