# Supplementary material for: Whole blood viscosity is associated with extrahepatic metastases and survival in patients with hepatocellular carcinoma
Source: PLoS One. 2021 Dec 2;16(12):e0260311. doi: 10.1371/journal.pone.0260311 (PMC8638904; doi:10.1371/journal.pone.0260311)
Supplement: S2 Table — (PDF) [file pone.0260311.s003.pdf]

S2 Table. Univariate and multivariate Cox regression analysis for factors associated with the future metastases among patients without initial metastases

	Univariate		Multivariate	
	HR (95 % CI)	p	HR (95 % CI)	p
PVTT	7.57 (2.82-20.33)	***<.001***	4.32 (1.52-12.22)	***.006***
Largest tumor size	1.14 (1.11-1.23)	***<.001***	1.11 (1.03-1.19)	***.004***
High diastolic WBV (> 16.0)	2.36 (0.97-5.79)	.060	2.12 (0.86-5.26)	.100

OR, odds ratio; CI, confidence interval; PVTT, portal vein tumor thrombosis; WBV, whole blood viscosity.
